# Dissection of the Genetic Basis of Rice Panicle Architecture Using a Genome-wide Association Study

**DOI:** 10.1186/s12284-021-00520-w

**Published:** 2021-09-06

**Authors:** Shaoxing Bai, Jun Hong, Ling Li, Su Su, Zhikang Li, Wensheng Wang, Fengli Zhang, Wanqi Liang, Dabing Zhang

**Affiliations:** 1 Joint International Research Laboratory of Metabolic and Developmental Sciences, State Key Laboratory of Hybrid Rice, School of Life Sciences and Biotechnology, Shanghai Jiao Tong University, Shanghai, 200240 China; 2 School of Agriculture, Food, and Wine, University of Adelaide, Adelaide, SA 5064 Australia; 3 Institute of Crop Sciences, Chinese Academy of Agricultural Sciences, Beijing, China; 4 Shenzhen Institute for Innovative Breeding, Chinese Academy of Agricultural Sciences, Shenzhen, China; 5 College of Agronomy, Anhui Agricultural University, Hefei, China

**Keywords:** Rice, Panicle architecture, Panicle length, Genome-wide association study, Quantitative trait loci, Hormone, Natural variation

## Abstract

Panicle architecture is one of the major factors influencing productivity of rice crops. The regulatory mechanisms underlying this complex trait are still unclear and genetic resources for rice breeders to improve panicle architecture are limited. Here, we have performed a genome-wide association study (GWAS) to analyze and identify genetic determinants underlying three panicle architecture traits. A population of 340 rice accessions from the 3000 Rice Genomes Project was phenotyped for panicle length, primary panicle number and secondary branch number over two years; GWAS was performed across the whole panel, and also across the japonica and indica sub-panels. A total of 153 quantitative trait loci (QTLs) were detected, of which 5 were associated with multiple traits, 8 were unique to either indica or japonica sub-panels, while 37 QTLs were stable across both years. Using haplotype and expression analysis, we reveal that genetic variations in the *OsSPL18* promoter significantly affect gene expression and correlate with panicle length phenotypes. Three new candidate genes with putative roles in determining panicle length were also identified. Haplotype analysis of *OsGRRP* and *LOC_Os03g03480* revealed high association with panicle length variation. Gene expression of *DSM2*, involved in abscisic acid biosynthesis, was up-regulated in long panicle accessions. Our results provide valuable information and resources for further unravelling the genetic basis determining rice panicle architecture. Identified candidate genes and molecular markers can be used in marker-assisted selection to improve rice panicle architecture through molecular breeding.

**Supplementary Information:**

The online version contains supplementary material available at 10.1186/s12284-021-00520-w.

## Background

Rice (*Oryza sativ*a L.) is a staple food crop. It has been estimated that an additional 116 million tons of rice per year will be needed by 2035 to feed growing populations (Seck et al. 2012). Panicle size and architecture are key agronomic traits that greatly affect yield, so understanding the molecular and genetic mechanisms underlying panicle development is of great importance to both plant biologists and modern breeders.

Panicle architecture has various quantitative characteristics, such as panicle length (PL), and the numbers of primary branches (PBN), secondary branches (SBN), and spikelets/grain, that affect yield (Crowell et al. 2016). Over the past two decades, a number of genes regulating panicle development have been identified and functionally characterized, e.g., *SP1* (Li et al. 2009), *DEP2* (Li et al. 2010; Zhu et al. 2010) and *DEP3* (Qiao et al. 2011) affect panicle length; and *OsSPL14* (Jiao et al. 2010; Miura et al. 2010), *ASP1* (Yoshida et al. 2012), and *APO1* (Terao et al. 2010) affect branch number. Many panicle trait genes are also pleiotropic, e.g., *MOC1* (Li et al. 2003), *LAX1* (Komatsu et al. 2001), and *OsSPL14* (Lu et al. 2013) affect not only branch number, but also tillering; while defects in *OsSPL18* decreased grain size and number and panicle length, but increased tillering (Yuan et al. 2019).

Panicle architecture QTLs commonly associate with other growth traits, in particular, heading date, e.g., *Hd1* (Zhang et al. 2019c), *EHD4* (Gao et al. 2013), *Ghd7* (Xue et al. 2008), *Ghd7.1* (Liu et al. 2013), and *DTH8* (Wei et al. 2010). Plant hormones can also play crucial roles in panicle development, and factors controlling hormone metabolism can affect panicle architecture, e.g., *Gn1a/OsCKX2* (Ashikari et al. 2005) and *LP/EP3* (Piao et al. 2009; Li et al. 2011) control spikelet number per panicle by regulating cytokinin; *OsGRF4* affects panicle architecture by mediating gibberellin and brassinosteroid responses (Che et al. 2015; Tang et al. 2018); while *OsPIN5b* determines panicle length by participating in auxin homeostasis (Lu et al. 2015).

With the advent of next-generation sequencing, genome-wide association studies (GWAS) that link genotype with phenotype in a natural population have rapidly become powerful gene/QTL mapping tools to detect complex agronomic traits, superseding the traditional time-consuming, imprecise QTL mapping techniques used in the past (Huang et al. 2010, 2012; Wang et al. 2018b). GWAS has been successfully used to dissect complex traits in multiple crop species, including barley (Cockram et al. 2010), maize (Wang et al. 2016), sorghum (Zhou et al. 2019), wheat (Luján Basile et al. 2019), potato (Okada et al. 2019), and cotton (Hinze et al. 2017). Dissection of QTL and identification of candidate genes associated with panicle development have recently been assisted by GWAS in rice. However, most of them have been conducted with low-density SNPs and do not have sufficient resolution to provide precise and complete information about the numbers and locations of the QTLs controlling the traits of interest (Reig-Valiente et al. 2018; Ta et al. 2018; Zang et al. 2015; Bai et al. 2016; Crowell et al. 2016; Rebolledo et al. 2016).

The recent 3000 Rice Genomes Project (3 K RGP) has provided a high-density single nucleotide polymorphisms (SNPs) database that is becoming popular for mining useful genetic information in rice using GWAS. For example, Jiang et al. (2020) detected 13 genetic loci related to development of leaf hairs; Zhang et al. (2019a,b,c) found 27 suggestively associated loci for sheath blight; while Shi et al. (2017) detected 22 significant salt tolerance-associated SNPs at the seed germination stage.

Here, we have used a panel of 340 diverse rice accessions selected from the 3 K RGP to perform GWAS on three panicle traits—PL, PBN, and SBN. We report 153 QTLs that significantly associate with variations in panicle architecture, and characterize the nucleotide diversity and expression levels of major *OsSPL18* haplotypes. In addition, we have used haplotype and gene expression analysis to identify three novel candidate genes (*OsGRRP*, *LOC_Os03g03480* and *DSM2*) that associate with panicle length. Our findings provide the basis for further elucidating mechanisms underlying panicle size and shape in rice.

## Results

### Panicle Phenotype Variation Across Rice Accessions

Panicle phenotype was analyzed in a panel of 340 japonica and indica accessions from the 3 K Rice Genomes Project with diverse genotypes, origins, and subpopulations. These rice varieties originate from 48 countries across Asia, Australia, North and South America, Europe, and Africa (Additional File 2: Table S1), and phylogenetic and principal component analyses confirmed that they fell into two main clades: indica (Xian, 161 accessions) and japonica (Geng, 179 accessions; Additional File 1: Figure S1a,b). The genome-wide linkage disequilibrium (LD) decay rates along chromosomes were found to be similar across the whole panel and indica and japonica sub-panels (Additional File 1: Figure S1c), and consistent with previous reports (Wang et al. 2018b).

**Fig. 1:**
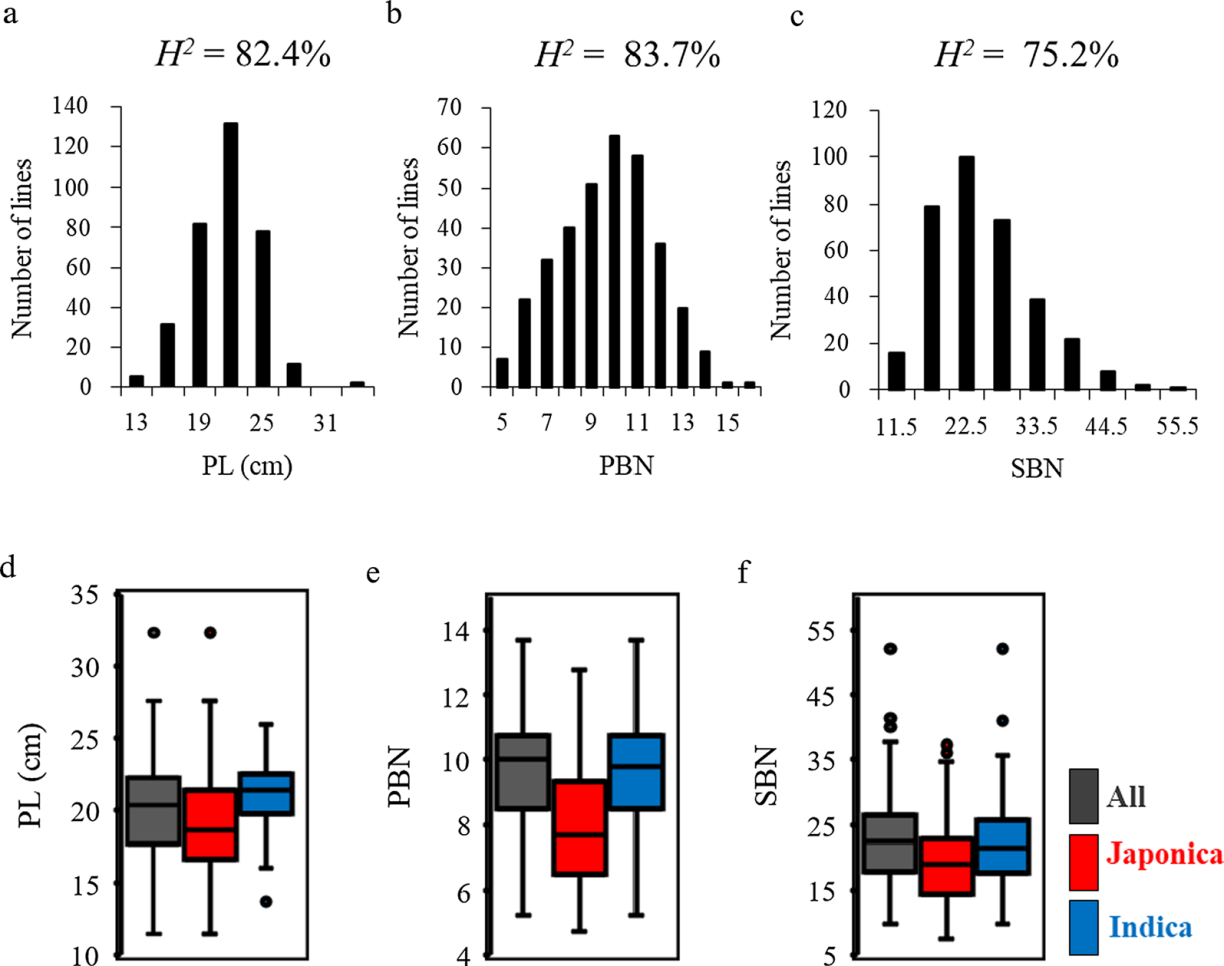
Panicle architecture traits of the three rice panels in 2017. **a**–**c** Distributions of **a** panicle length (PL), **b** primary branch number (PBN), and **c** secondary branch number (SBN) of the whole panel. *H*^2^, broad-sense heritability. **d**–**f** Box plot of the phenotypic variation of **d** PL, **e** PBN, and **f** SBN within the whole panel, and indica and japonica sub-panels. Boxes show median, and upper and lower quartiles. Whiskers extend to 1.5 × the interquartile range, with any remaining points indicated with dots

Panicle length (PL), primary branch number (PBN), and secondary branch number (PBN) for the main panicle in all 340 varieties were evaluated for 2 growing seasons (2015 and 2017 in Hainan, China; Table 1). SBN displayed the largest phenotypic variation, with coefficients of variance (CVs) from 30–35%, while the PL and PBN traits were less variable. While all three traits exhibited normal distribution (Fig. 1a–c, Additional File 1: Figure S2a–c), there was an obvious difference between the two years. Panicles in 2015 were generally longer and more branched (Table 1), indicating that these quantitative traits are controlled by genetic and environmental factors. In general, panicle architecture traits showed a high heritability, with broad-sense heritability (*H*^*2*^) from 75 to 84% in 2017 samples. These populations thus exhibited extensive variation in panicle architecture traits strongly linked to genotype, making them suitable for genome-wide association studies (GWAS).

**Table 1:** Phenotypic variations of panicle traits

Trait	2015				2017			
	Mean	Min	Max	CV	Mean	Min	Max	CV
Panicle length (cm)	22.1 ± 3.3	13.5	37.1	15	20.1 ± 3.2	11.5	32.4	16
Primary branches (#)	10.7 ± 2.0	6.0	17.0	19	9.0 ± 2.2	4.8	16.0	24
Secondary branches (#)	29.0 ± 9.0	8.0	61.0	30	22.0 ± 7.8	7.6	52.0	35

Branch numbers per panicle; mean ± SD of 3 replicates

*CV* coefficient of variation (%)

Analysis of the japonica and indica sub-panels revealed that values for the three panicle traits largely overlapped, though values for indica varieties were generally higher than for japonica varieties (Fig. 1d–f, Additional File 1: Figure S2d–f), consistent with previous reports (Ta et al. 2018). The three panicle traits only weakly correlated with one another in each year (Additional File 1: Figure S3a, b). Generally, correlations were strongest between PBN and SBN, as secondary branches originate from primary branches; with the next highest correlation between PL and PBN, again, as primary branches come from the rachis of the panicle.

### Association Mapping of Three Panicle Architecture Traits

**Fig. 2:**
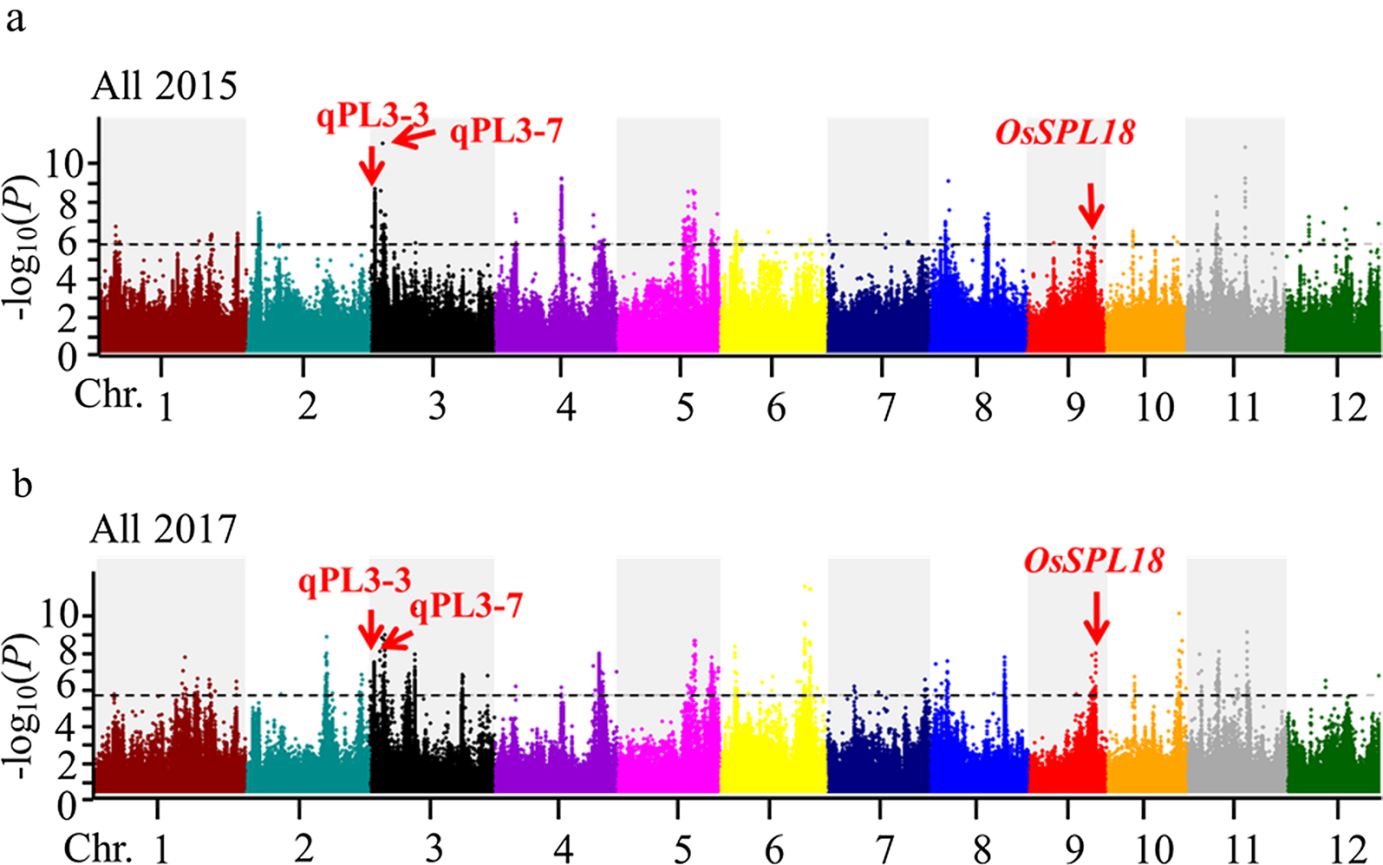
Manhattan plots of GWAS for panicle length in **a** 2015 and **b** 2017 for all accessions. The red arrows represent three significant associations, assigned when the peak SNP correlation exceeds the significance threshold, −log_10_(*P*) > 5.69 (*P* < 2.04 × 10^–6^)

Across the three traits in two years, 1087 significant SNPs yielded 153 QTLs in 148 genomic regions across the three rice panels (Fig. 2, Additional File 1: Figures S4–S6 and Additional File 2: Table S2). GWAS mapping resulted in 129 individual QTLs (115 for PL, 12 for PBN, and 2 for SBN) for the whole panel; 11 QTLs (7 for PL, 1 for PBN, and 3 for SBN) for the japonica sub-panel; and 21 QTLs (6 for PL, 6 for PBN, and 9 for SBN) for the indica panel. Only 5 QTLs (qPL3-14/qPBN3-1, qPL5-3/qPBN5-1, qPL5-16/qPBN5-2, qPL6-1/qPBN6-1, qPL11-3/qSBN11-1) were common across more than one trait. Intriguingly, there were no common QTLs between PBN and SBN, suggesting different regulatory mechanisms underlie these two levels of panicle branching. Only 37 QTLs (31 for PL and 6 for SBN) were stable across 2015 and 2017, suggesting that these loci contain genes that function in panicle architecture without the influence of environmental factors (Additional File 2: Table S3).

Among the 37 stable QTLs, comparison with previous literature revealed overlaps with 8 known genes, and 10 QTLs from other GWAS analyses and QTARO (Additional File 2: Tables S3 and S4, respectively). The remaining 19 QTLs (15 for PL and 4 for SBN) are the novel, and thus we did further analysis (Additional File 2: Table S5).

### *OsSPL18*, the Candidate Gene of qPL9-6

**Fig. 3:**
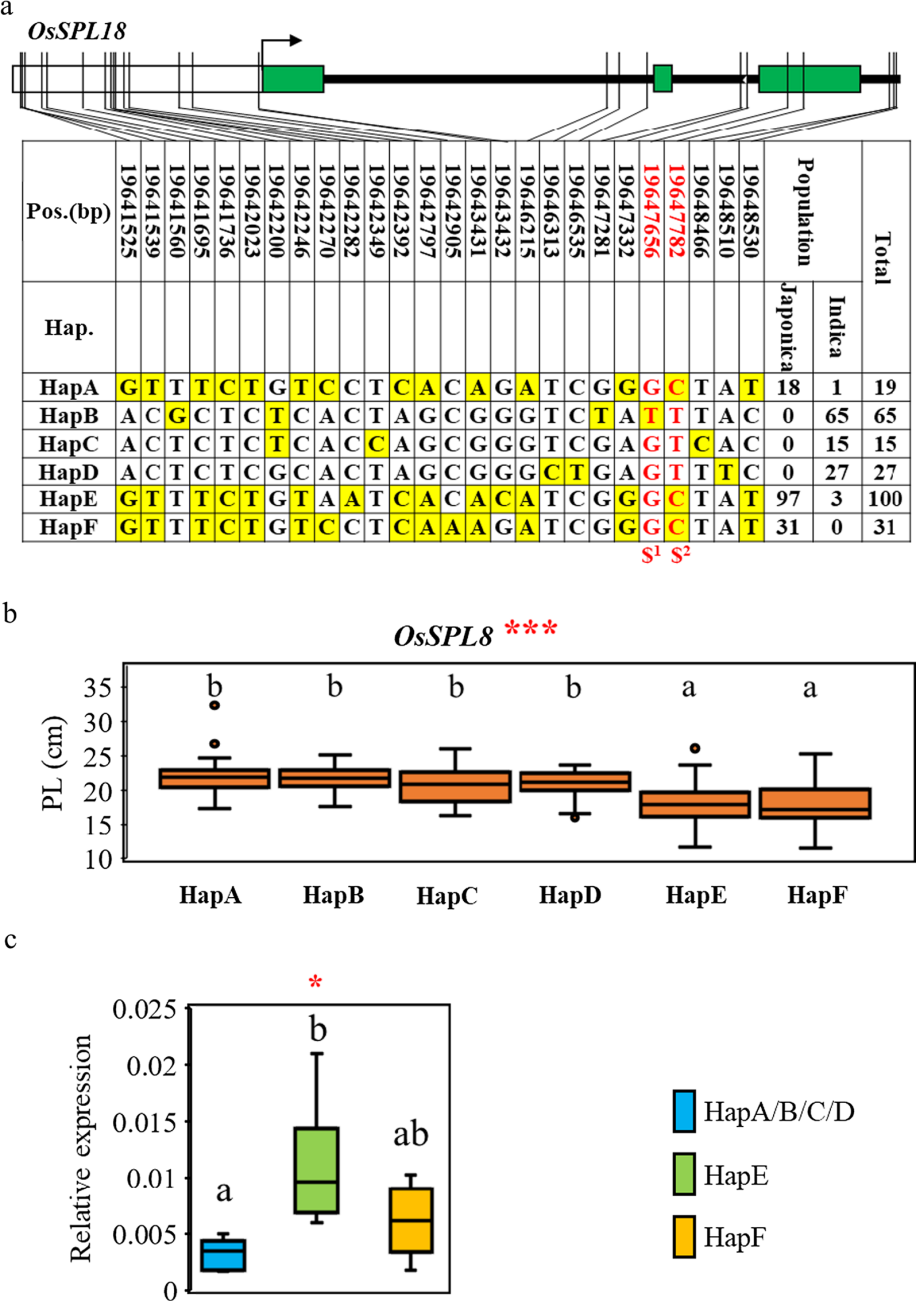
Haplotype analysis of *OsSPL18*. **a** Six haplotypes of *SPL18* based on 26 SNPs observed in all assessed rice accessions. The schematic representation of *SPL18* gene structure (upper) shows the promoter as a white box, exons as green boxes, and introns and intergenic regions as black lines. Thin black lines indicate the genomic position of each SNP. Haplotypes with fewer than 10 accessions are not shown. Yellow highlight indicates SNP alternatives. The SNP in red and bold is a non- synonymous SNP. ‘$^1^’ indicates a missense mutation from Gly to Val. ‘$^2^’ indicates a missense mutation from Thr to Met **b–c** Box plots for **b** panicle length based on the six haplotypes for *SPL18* in 2017 and **c** *OsSPL18* expression levels in different haplotypes relative to *OsActin1*. Boxes show median, and upper and lower quartiles. Whiskers extend to 1.5 × the interquartile range, with any remaining points indicated with dots. *P < 0.05, ***P < 0.001 (ANOVA). Letters indicate significant differences, *P* < 0.05 (Duncan’s multiple comparison test)

One stable QTL, qPL9-6, was found to contain *OsSPL18* gene, which had previously been reported to affect panicle length and grain number via new regulatory pathways (Yuan et al. 2019); but the influence of genetic sequence diversity within this gene was not examined. We analyzed SNPs in the 2 kb upstream promoter and genomic *OsSPL18* coding region to reveal six major haplotypes. Indica varieties generally contained a higher proportion of HapB, HapC, and HapD types, while japonica varieties contained a higher proportion of HapA, HapE, and HapF sequences (Fig. 3a). The panicle length of accessions containing HapE and HapF was significantly lower than for other haplotypes (Fig. 3b). Therefore, this result is useful for clarifying the panicle length variation within japonica varieties because haplotype E and F exhibited smaller panicle length than other haplotypes and were only found in *japonica* group except three *indica* accessions (Fig. 3a).

A large proportion of SNPs were identified in the promoter and introns compared with the coding sequence, suggesting that these genetic variations may impact *OsSPL18* gene expression. Gene expression analysis from rachis revealed that HapE varieties had significantly higher levels of *OsSPL18* expression than HapA/B/C/D varieties; HapF expression levels were intermediate, and not significantly different to either of the other groups (Fig. 3c, Additional file 2: Table S6). In addition, haplotype analysis showed that two non-synonymous SNPs variation (Chr9_19647656, base G-T, amino acid Gly-Val, Chr9_19647782, base C-T, amino acid Thr-Met) were also significantly associated with panicle length (data not shown). Therefore, both polymorphisms in the coding and promotor regions of *OsSPL18* may cause the variation of panicle length.

### *OsGRRP,* the Candidate Gene of qPL3-7

**Fig. 4:**
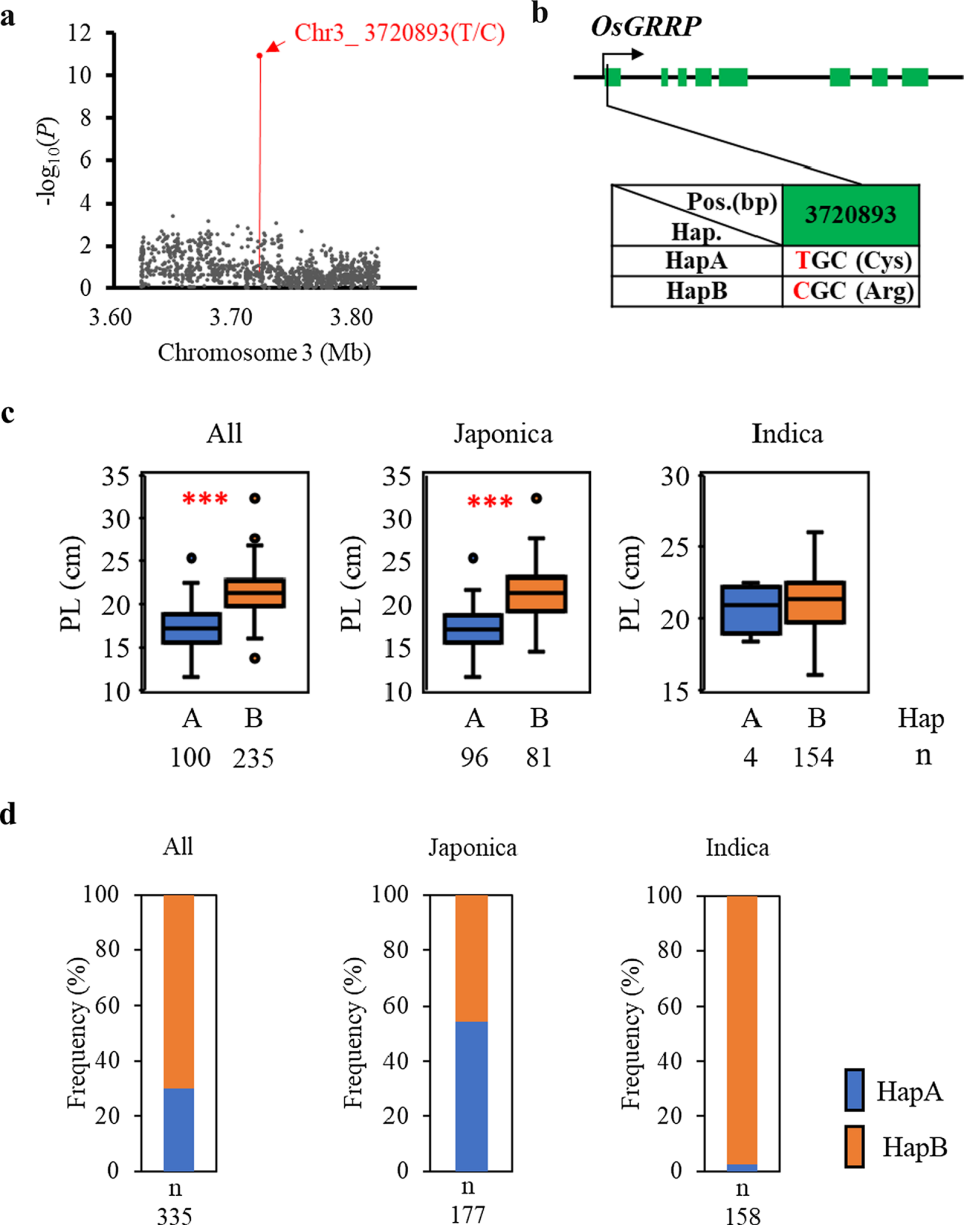
Haplotype analysis of the candidate gene in qPL3-7. **a** Local Manhattan plot of qPL3-3 on chromosome 3. The red arrow indicates the position of the lead SNP. **b** Schematic representation of *OsGRRP* gene structure and the position of peak SNP used for haplotype analysis. Green boxes indicate exons. **c** Box plots for panicle length (PL) in the two haplotypes of *OsGRRP* in all, japonica, and indica accessions in 2017. Number of accessions (n) of each haplotype (Hap) in each panel given under the x-axis. Boxes show median, and upper and lower quartiles. Whiskers extend to 1.5 × the interquartile range, with any remaining points indicated with dots. ***P < 0.001 (Welch two sample *t*-test). **d** Frequencies of the two *OsGRRP* haplotypes in all, japonica, and indica accessions. n, number of accessions in each panel

The lead SNP (Chr3_3720893(T/C), *P* = 1.1 × 10^–11^ in 2015 and 1.19 × 10^–09^ in 2017) in qPL3-7 caused a missense mutation from cysteine to arginine in the first exon of *OsGRRP* (*LOC_Os03g07310*), predicted to encode a growth regulator–related protein (Fig. 4a–b). Panicle length correlated significantly with gene haplotype at this location in the whole panel and japonica sub-panel in both years. In the indica sub-panel, there was no difference in the panicle length of two haplotype (Fig. 4c, Additional file 1: Figure S7). However, an obvious difference in allele frequency was observed between the japonica and indica varieties: nearly all (97%) of the indica lines contained the long panicle HapB allele, compared with 46% of the japonica varieties (Fig. 4d). This genetic difference may partially explain the longer average panicle length in indica varieties (Ta et al. 2018).

### Two candidate Genes of qPL3-3

qPL3-3 was especially attractive, as it contained the highest number of SNPs (444 and 291 in 2015 and 2017, respectively; Additional File 2: Table S5). After removal of genes encoding hypothetical proteins, retrotransposons, and transposon proteins, 28 candidate genes were identified (Additional File 2: Table S7).

**Fig. 5:**
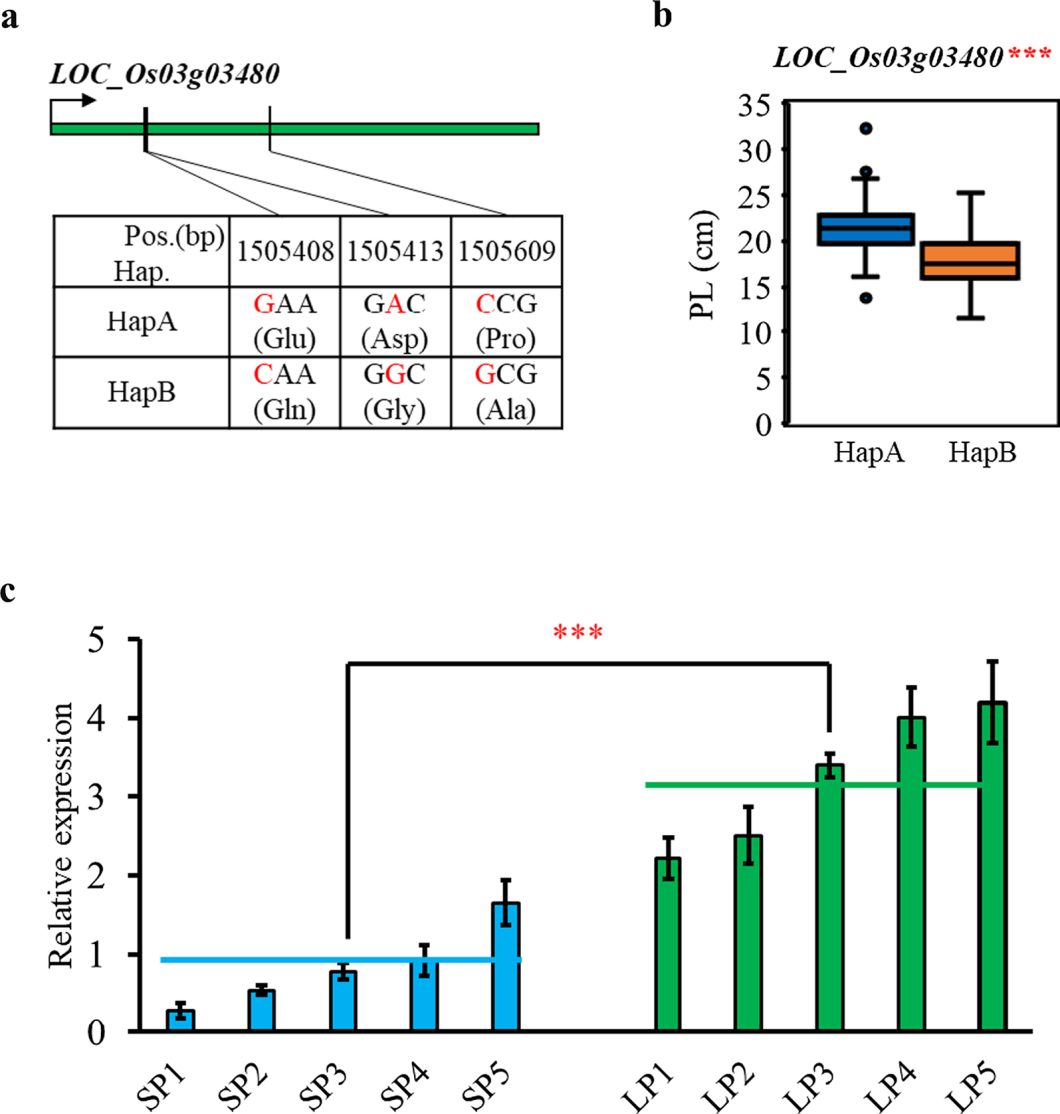
Two candidate genes analysis in qPL3-3. **a** Schematic representation of *LOC_Os03g03480* structure and the position of 3 non-synonymous SNP used for haplotype analysis. **b** Box plots for panicle length (PL) in the two haplotypes of *LOC_Os03g03480* in 2017. Boxes show median, and upper and lower quartiles. Whiskers extend to 1.5 × the interquartile range, with any remaining points indicated with dots. ***P < 0.001 (Welch two sample *t*-test). **c** Relative expression of *DSM2* in short panicle (SP) and long panicle (LP) accessions. The blue and green horizontal lines depict the average expression levels in the SP and LP accessions, respectively. ***P < 0.001 (Welch two sample *t*-test). SP1: IRIS_313-8085; SP2: IRIS_313-8096; SP3: IRIS_313-8099; SP4: IRIS_313-8195; SP5: IRIS_313-8048; LP1: IRIS_313-8903; LP2: B199; LP3: IRIS_313-9505; LP4: IRIS_313-7993; LP5: IRIS_313-799

We observed 23 stable non-synonymous SNPs in the coding region of 6 genes detected in two years (Additional File 2: Table S7). *LOC_Os03g03480*, encoding a DUF623 domain containing protein caught more attention. Previous studies have demonstrated that these kinds of proteins are involved in panicle length (Schmitz et al. 2015; Yang et al. 2018). Two types of haplotypes based on 3 non-synonymous SNPs and panicle length correlated significantly with gene haplotypes (Fig. 5a, b), suggesting that *LOC_Os03g03480* might be a candidate gene involved in panicle length. Besides these 6 genes, *OsSWN5* and *LOC_Os03g03260* in qPL3-3 were of interest as other genes annotated as “no apical meristem protein” and “homeobox domain containing protein” (*LOC_Os01g47710* and *LOC_Os05g48990*) had previously been shown to have a role in controlling panicle length in rice (Wang et al. 2014; Sakuraba et al. 2015; Fang et al. 2020), so *OsSWN5* and *LOC_Os03g03260* in qPL3-3 were of interest. Similarly, panicle length is sensitive to genes involved in plant hormone biosynthesis or signaling, including ABA (Wang et al. 2015b; Hong et al. 2016; Zhang et al. 2016), so *DSM2* (*LOC_Os03g03370*), which encodes a β-carotene hydroxylase involved in the biosynthesis of the ABA precursor zeaxanthin (Du et al. 2010), was also a gene of interest in qPL3-3. These 3 genes were thus selected as candidates for expression analysis in a small group of 5 short panicle (SP) and 5 long panicle (LP) accessions (Fig. 5b, Additional file 1: Figure S8). Only *DSM2* was differentially expressed between SP and LP varieties, being more highly expressed in the LP rather than in SP lines. Thus, expression of *DSM2* was positively associated with panicle length, and also likely accounts for the association of qPL3-3 with panicle length.

## Discussion

Rice panicle size and architecture are important factors for rice yield. Panicle architecture is a complex quantitative trait controlled by both genes and environment (Cubry et al. 2020). The diversity of genetic variation in natural populations is an important potential source of beneficial alleles. Here, we have performed GWAS on 340 rice accessions selected from 3 K RGP to identify new genes underlying panicle architecture traits.

### Phenotypic Variations for Targeted Traits

A high heritability was observed in the three analyzed panicle architecture traits, indicating that their diversity was more under genetic than environmental control in these accessions (Fig. 1). Moreover, similar trends of positive correlation between these three traits were observed in the whole panel and indica and japonica sub-panels (Additional File 1: Figure S3), consistent with the GWAS results in which some QTLs could be detected in two years or associated with multiple traits (Additional File 2: Table S2). However, despite the high correlation between PBN and SBN, there was no single SNP that associated with both PBN and SBN, suggesting that the mechanisms related to their establishment and function are regulated by discrete genetic determinants (Ta et al. 2018).

A small number of GWAS sites in this study were detected only in indica or japonica sub-panels (Additional File 2: Table S2). Differences between panicle architecture between japonica and indica subspecies have previously been observed, driven in part by artificial human selection and geographic regions of cultivation, such as *Ghd7*, evolved from two distinct ancestral gene pools (Xue et al. 2008). Another gene from japonica cultivars, *LSCHL4* (allelic to *NAL1*), was shown to enhance grain productivity when introduced into indica cultivars through pleiotropic effects on plant architecture (Zhang et al. 2014). These results suggest that there are different genetic networks regulating panicle architecture variation in indica and japonica sub-panels.

### GWAS-Based Mapping of PL, PBN, and SBN QTLs

GWAS is an efficient technique to analyze genetic variation for multiple traits in rice. Several studies employed GWAS in the rice population for various panicle architecture traits and reported some novel QTLs (Zhang et al. 2015; Sahu et al. 2020), but such study in diverse rice landrace is limited. In the present study, our results yielded 153 QTLs for panicle architecture traits. Of these, 5 were associated with more than one trait, which suggests that these loci contain pleiotropic genes (Additional File 2: Table S2). 37 QTLs could be detected over 2 years (Additional File 2: Table S3), indicating these QTLs in rice were stable against environmental factors and supporting the notion of panicle architecture depends not only on genetic diversity but also the environmental factors. Among the 37 stable QTLs, we observd that 8 genes were known to be involved in panicle architecture (Additional File 2: Table S3), suggesting that our results are of high reliability. For example, *SPOTTED LEAF3*(SPL3), which is involved in ABA and ethylene signalling pathways (Wang et al. 2015a), was co-localized with qPL3-6. *LAX2,* which is involved in maintenance of apical meristems in rice, including in the panicle (Tabuchi et al. 2011), was found in qPL4-3. 10 QTLs from other studies and the remaining 19 QTLs are reported here for the first time, and represent promising targets for further analysis of panicle architecture (Additional File 2: Table S5). Overall, the QTLs detected in this study indicated that a number of as yet unknown factors may be involved in the determination of panicle architecture and helpful in molecular marker-assisted selection of rice panicle shape breeding.

### Candidate Gene Analysis for Three QTLs

In rice, the SQUAMOSA-PROMOTER BINDING PROTEIN-LIKE (SPL) family proteins play important roles during panicle development. To date, five known *SPL* genes (*OsSPL6, OsLG1/OsSPL8, OsSPL13*, *IPA1/WFP/OsSPL14*, and *OsSPL18*) have been reported to regulate panicle architecture in rice (Jiao et al. 2010; Miura et al. 2010; Ishii et al. 2013; Zhu et al. 2013; Si et al. 2016; Wang et al. 2018a; Yuan et al. 2019). *OsSPL18* was located in qPL9-6, a QTL associated with panicle length, and we have used haplotype and expression analysis to show that nucleotide polymorphisms in the *OsSPL18* promoter significantly affect gene expression and panicle length (Fig. 3). Haplotypes with the desired phenotype may be suitable for traditional rice breeding and are also good targets for molecular marker-assisted selection breeding.

Analysis of the leading SNP in *OsGRRP* in qPL3-7 revealed two haplotypes (Fig. 4a, b). Significant differences in mean panicle length were associated with haplotype in the whole panel and japonica accessions, with HapB directing the longer phenotype (Fig. 4c), indicating that *OsGRRP* may be responsible for panicle length variation. Differences in haplotype frequency in the two subspecies, with 97% of indica accessions containing HapB (Fig. 4d), suggests that *OsGRRP* has been subjected to selection during rice breeding and may have contributed to differentiation of the two subspecies.

Among the QTLs for panicle length that were the same across both years, we selected qPL3-3 for further analysis of causal genes due to high SNP concentrations (Additional File 2: Table S5). We, firstly, based on functional annotation and haplotype analysis, proposed that *LOC_Os03g03480,* a DUF623 domain containing protein, is one of candidate gene in qPL3-3. Furthermore, three candidate genes were selected for expression analysis based on their functional annotation (Additional File 2: Table S7). Expression of *DSM2*, a fatty acid hydroxylase involved in ABA synthesis, was found to correlate with panicle length (Fig. 5), indicating that during the rice panicle development, the altered expression of enzyme genes related to the ABA synthesis pathway contributes to the rice panicle length determination, consistent with the previous results (Wang et al. 2015b; Zhang et al. 2016).

### Natural Variations of Candidate Genes

Natural variation in genes involved in the regulation of important agronomic traits has been utilized by breeders to improve rice yield. Although dozens of genes related to panicle architecture have been identified using multiple strategies, only a few reported genes, like *NAL1*, have been applied in rice breeding (Fujita et al. 2013; Zhang et al. 2014).

GWAS enables the exploration of the large of alleles present in genetic resources and that are useful in rice improvement. The differential expression profile strategy combination of GWAS is a powerful method for target-gene mining. In this study, Expression analysis of candidate genes revealed two genes, *DSM2* and *OsSPL18*, that were up- and down-regulated, respectively, in long panicle accessions. These results suggest that transcriptional regulation of the candidate genes may through SNP variations in promoter region and then contribute to panicle length variation in rice. Modification of the core promoters of targeted genes by genome editing is a widely applicable and reliable approach for the fine-tuning of the expression of target genes (Huang et al. 2020; Zeng et al. 2020), so the further studies to understand the mechanisms involved in transcriptional regulation of these two genes will be conducted. Moreover, haplotype analysis was carried out for two genes (*OsGRRP* and *LOC_Os03g03480*) using significant non-synonymous SNPs located inside of the gene CDS region, especially a non-synonymous SNP (Chr3_3720893(T/C)) variation was observed in *OsGRRP*, which resulted in an amino-acid substitution from cysteine to arginine (Fig. 4), was most likely to be the functional site. Overall, further functional analysis of these candidate gene will help us better understand the genetic basis for natural variation in rice panicle architecture control and suggest that these genes may be used to improve the rice yield.

## Conclusions

In our study, we have dissected the genetic basis underlying differences in rice panicle architecture traits in indica and japonica subspecies. We identified natural variation in the promoter of *OsSPL18* that affects gene expression level and panicle length. Using gene functional annotation, haplotype analysis, and expression analysis after GWAS, we have identified three novel candidate genes associated with panicle length. Genetic complementation, overexpression, and knockout studies of these three candidates will clarify their role in directing rice panicle length, and will be a focus for further studies. More broadly, other SNPs and genes reported here could be used for future research, gene validation, and marker-assisted selection for molecular breeding of rice with enhanced panicle architecture.

## Materials and Methods

### Plant Materials and Growing Conditions

A panel of 340 rice varieties were selected from the 3 K Rice Genomes Project, comprising 161 indica (Xian) and 179 japonica (Geng) accessions (Wang et al. 2018b). Detailed information regarding these accessions, including their geographical origin, is shown in Additional File 2: Table S1. All accessions were grown in the experimental fields around Sanya (Hainan province, China; 18°15′N, 109°30'E) from January to April in 2015 and 2017. Six plants of each variety were planted in a row with 17 cm between plants and 20 cm between rows. Panicle traits were measured only from the main tiller in each plant.

### Population Genetic Analyses

3,214,392 SNPs (all accessions), 923,587 SNPs (japonica accessions), and 1,501,869 SNPs (indica accessions) with a minor allele frequency (MAF) of ≥ 5% and a missing rate of ≤ 20% were selected for population and association analyses.

K-estimation (K = 2) was based on the subgroups of all accessions(Wang et al. 2018b). PCA were performed with GCTA software (https://cnsgenomics.com/software/gcta/#Overview) and neighbor-joining trees were constructed with SNPhylo based on SNPs in all accessions (Lee et al. 2014).

Linkage disequilibrium (LD) measures between SNP loci at the individual chromosome level were plotted by evaluating r^2^ (squared allele frequency correlation) estimators between significant marker(s) using the PopLDdecay (Zhang et al. 2019a).

### Data Analysis

The three panicle related traits were measured as previously described (Crowell et al. 2016). Statistical analyses were performed in Excel 2010 (Microsoft) and SPSS software (version 18.0). Welch two sample *t*-test were used to analyze differences between the three rice panels. Panicle length and expression data of *OsSPL18* haplotypes were analyzed using ANOVA and Duncan’s multiple test in the SPSS software.

Pearson’s correlations were used to examine the correlations between traits in 2015 and 2017, and the broad-sense heritability ($H_{B}^{2}$) was calculated in 2017 to describe how each trait was affected by the environment as follows: $H^{2} = var_{\left( G \right)} /\left( {var_{\left( G \right)} + var_{\left( E \right)} } \right),$ where $var_{\left( G \right)}$ and $var_{\left( E \right)}$ are the genotypic and experimental variance, respectively.

### GWAS for Panicle Architecture

The 3 K RGP 4.8mio SNP dataset was downloaded from the Rice SNP-Seek Database (http://snp-seek.irri.org/; Alexandrov et al. 2015). GWAS was performed on SNPs as described above using the factored spectrally transformed linear mixed models (FaST-LMM; Lippert et al. 2011). The significance threshold for the identification of QTLs was set to *P* < 2.04 × 10^–6^, and the SNP with the minimum *P* value was considered the lead SNP. Manhattan plots for the GWAS results were drawn using the R package ‘qqman’ (https://www.r-project.org/).

### Haplotype Analysis and Identification of Candidate Genes

Candidate genes were scanned within the 200 kb region centered on the lead SNP of each QTL (using the reference Nipponbare genome (http://rice.plantbiology.msu.edu/cgi-bin/gbrowse/rice/). The 2019 QTARO and MSU databases (http://qtaro.abr.affrc.go.jp and http://rice.plantbiology.msu.edu) were used to identify previously reported QTLs/genes present in the LD region. Candidate genes were identified based on predicted function from the rice genome annotation project.

All SNPs in the promoter region (2 kb) of *OsSPL18* and non-synonymous SNPs in *OsSPL18* exons, introns, and 3′ untranslated region were selected from the Rice SNP-Seek Database (https://snp-seek.irri.org/).

### Gene Expression Analysis

Rachis from 5 short panicle (SP) and 5 long panicle (LP) accessions, grown in the paddy field of Shanghai JiaoTong University, were collected in triplicate at the stage of 50% panicle emergence from the leaf sheath based on the PL in 2017. Average PL for SP accessions was 12.4 cm; for LP accessions, 25.9 cm in 2017.

Total RNA was extracted using the Trizol reagent (Invitrogen), following the manufacturer’s instructions. cDNA was synthesized from total RNA using the FastQuant RT Kit (with gDNase; Tiangen). Quantitative real time PCR (qRT-PCR) was performed in a two-step reaction using SuperReal PreMix Color (SYBR Green; Tiangen) on a Roche Light Cycler 2.10 system using the 2^−ΔΔCt^ method (Livak and Schmittgen 2001) with three technical replicates. Expression levels were normalized to *OsActin1* (LOC_Os03g50885).

The sequences of the candidate genes were downloaded from the Rice Genome Annotation Project (http://rice.plantbiology.msu.edu/analyses_search_locus.shtml). Primer sequences for candidate genes were downloaded from the qPrimerDB-qPCR Primer Database (https://biodb.swu.edu.cn/qprimerdb/best-primers-ss) except for *OsSWN5*, for which primers were designed by NCBI. Primer sequences are given in Additional File 2: Table S8.

## Supplementary Information

**Additional file 1**.** Figure S1**: Population structure of 340 rice accessions, comprising 161 indica and 179 japonica varieties. ** Figure S2**: Panicle architecture traits of the three rice panels in 2015. ** Figure S3**: Correlations between PL, PBN, and SBN from each GWAS panel in 2015 and 2017. ** Figure S4**: Manhattan plots of GWAS for panicle length. ** Figure S5**: Manhattan plots of GWAS for primary branch number. ** Figure S6**: Manhattan plots of GWAS for secondary branch number. ** Figure S7**: Panicle length in the two haplotypes of OsGRRP in 2015. ** Figure S8**: Relative expression of OsSWN5 and LOC_Os03g03260 in SP and LP accessions.

**Additional file 2**.** Table S1**: Association panel accessions and phenotypes.** Table S2**: Significantly associated loci based on GWAS for PL, PBN and SBN in the whole panel, and the japonica and indica sub-panels, grown in 2015 and 2017.** Table S3**: 37 QTLs stable across 2015 and 2017.** Table S4**: Colocalization of detected associations with known GWAS-derived associations and the QTARO database.** Table S5**: 19 newly identified QTLs in our study.** Table S6**: Accessions of different OsSPL18 haplotypes for whole panel and qRT-PCR.** Table S7**: Genes in qPL3-3.** Table S8**: Primers used in this study.

## Data Availability

All data generated or analyzed during this study are included in this published article and its supplementary information files.

## Abbreviations

ABA: Abscisic acid
CV: Coefficient of variance
GWAS: Genome-wide association study
*H*^*2*^: Broad-sense heritability
Hap: Haplotype
LP: Long panicle
n: Number
PBN: Primary branch number
PL: Panicle length
QTL: Quantitative trait locus
SBN: Secondary branch number
SNP: Single nucleotide polymorphism
SP: Short panicle
