# Supplementary material for: Dissection of the Genetic Basis of Rice Panicle Architecture Using a Genome-wide Association Study
Source: Rice (N Y). 2021 Sep 6;14:77. doi: 10.1186/s12284-021-00520-w (PMC8421479; doi:10.1186/s12284-021-00520-w)
Supplement: Supplementary file 1 — Additional file 1. Figure S1: Population structure of 340 rice accessions, comprising 161 indica and 179 japonica varieties. Figure S2: Panicle architecture traits of the three rice panels in 2015. Figure S3: Correlations between PL, PBN, and SBN from each GWAS panel in 2015 and 2017. Figure S4: Manhattan plots of GWAS for panicle length. Figure S5: Manhattan plots of GWAS for primary branch number. Figure S6: Manhattan plots of GWAS for secondary branch number. Figure S7: Panicle length in the two haplotypes of OsGRRP in 2015. Figure S8: Relative expression of OsSWN5 and LOC_Os03g03260 in SP and LP accessions. [file 12284_2021_520_MOESM1_ESM.docx]

**
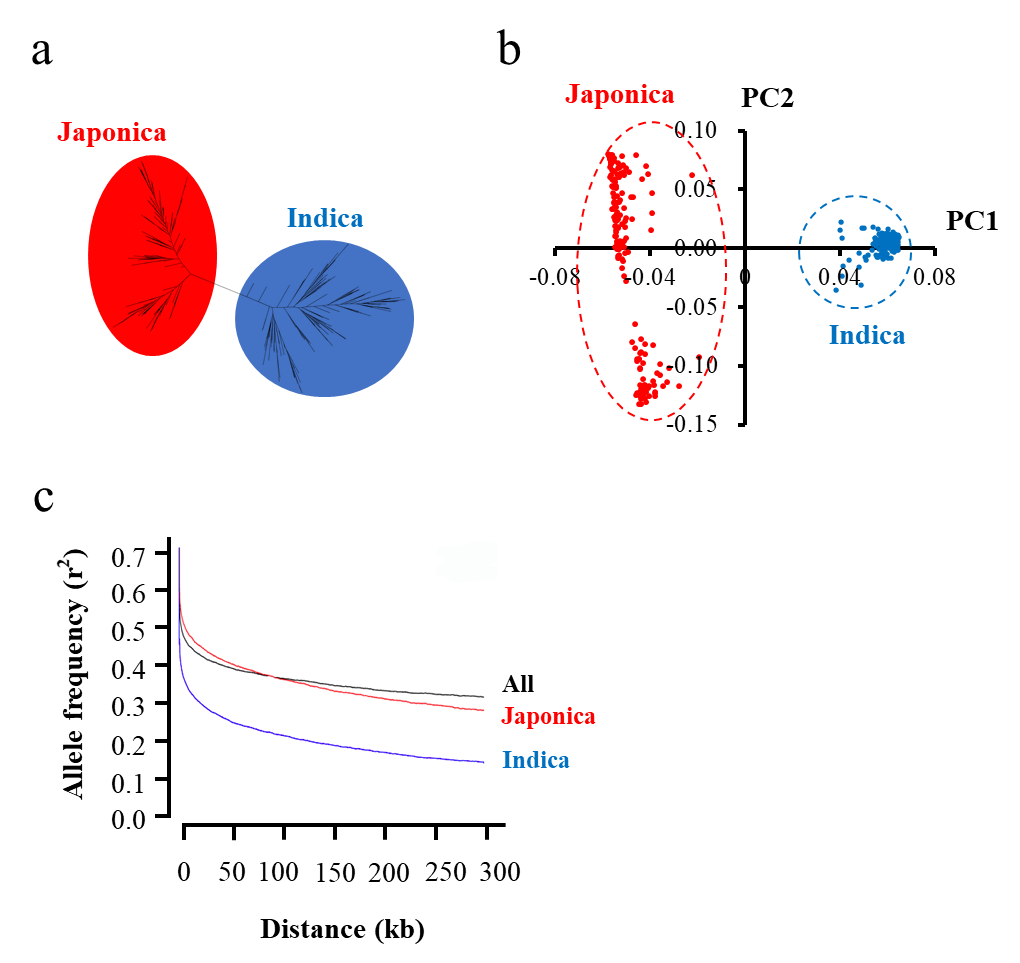
**

**Figure S1** Population structure of 340 rice accessions, comprising 161 indica and 179 japonica varieties. **a** Neighbor-joining tree based on all the SNPs from 340 rice accessions. **b** Principal component analysis of the genotypic data. **c** Genome-wide average linkage disequilibrium decay for three populations using the SNP data. When allele frequency (r^2^) decays to half of its maximum value, the corresponding physical distance is recorded as the linkage disequilibrium (LD) attenuation distance of the whole genome.


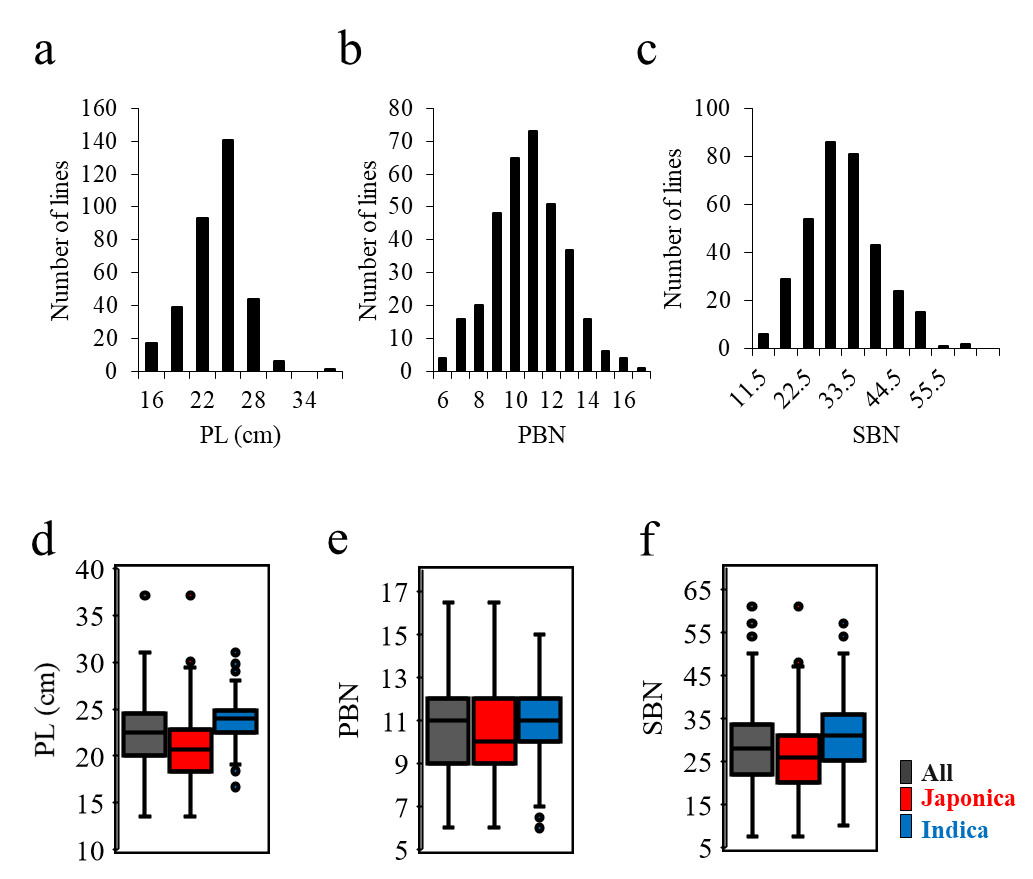


**Figure S2** Panicle architecture traits of the three rice panels in 2015. **a**–**c** Distributions of **a** panicle length (PL), **b** primary branch number (PBN), and **c** secondary branch number (SBN) of the whole panel. **d**–**f** Box plot of the phenotypic variation of **d** PL, **e** PBN, and **f** SBN within the whole panel, and indica and japonica sub-panels. Boxes show median, and upper and lower quartiles. Whiskers extend to 1.5×the interquartile range, with any remaining points indicated with dots.


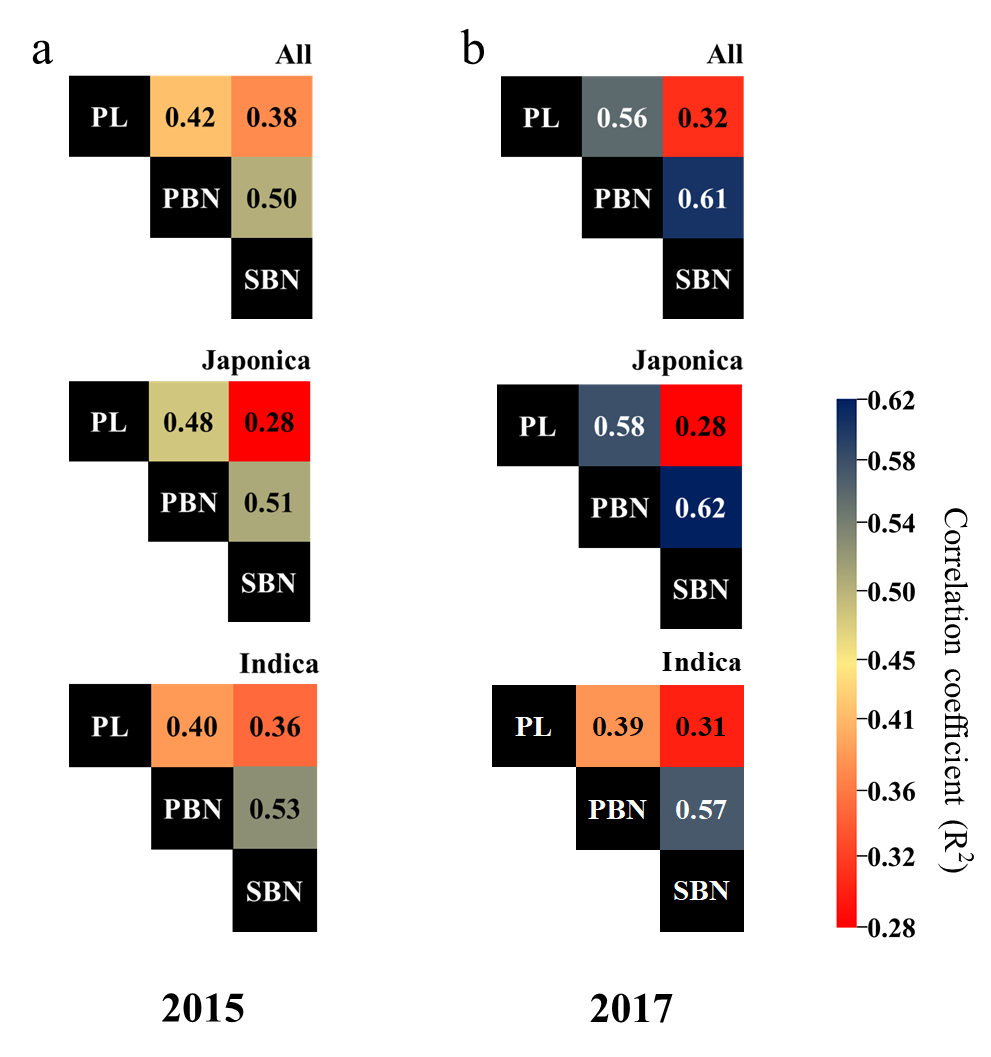


**Figure S3** Correlations between PL, PBN, and SBN from each GWAS panel in **a** 2015 and **b** 2017


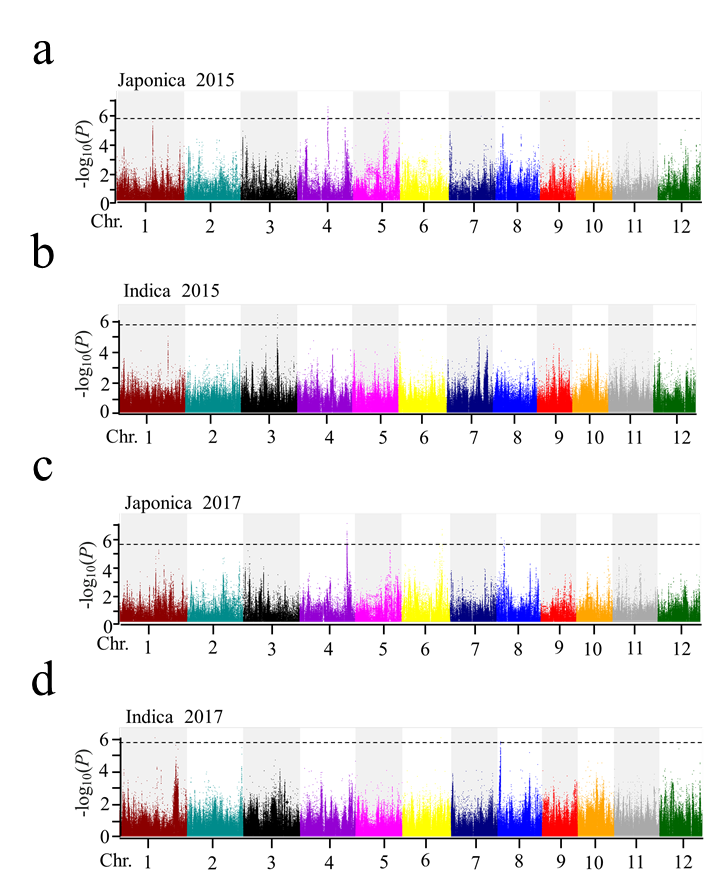


**Figure S4** Manhattan plots of GWAS for panicle length in **a–b** 2015 and **c–d** 2017 for **a, c** japonica accessions; and **b, d** indica accessions. The black horizontal dashed line indicates the genome-wide significance threshold, -log_10_(*P*) > 5.69.


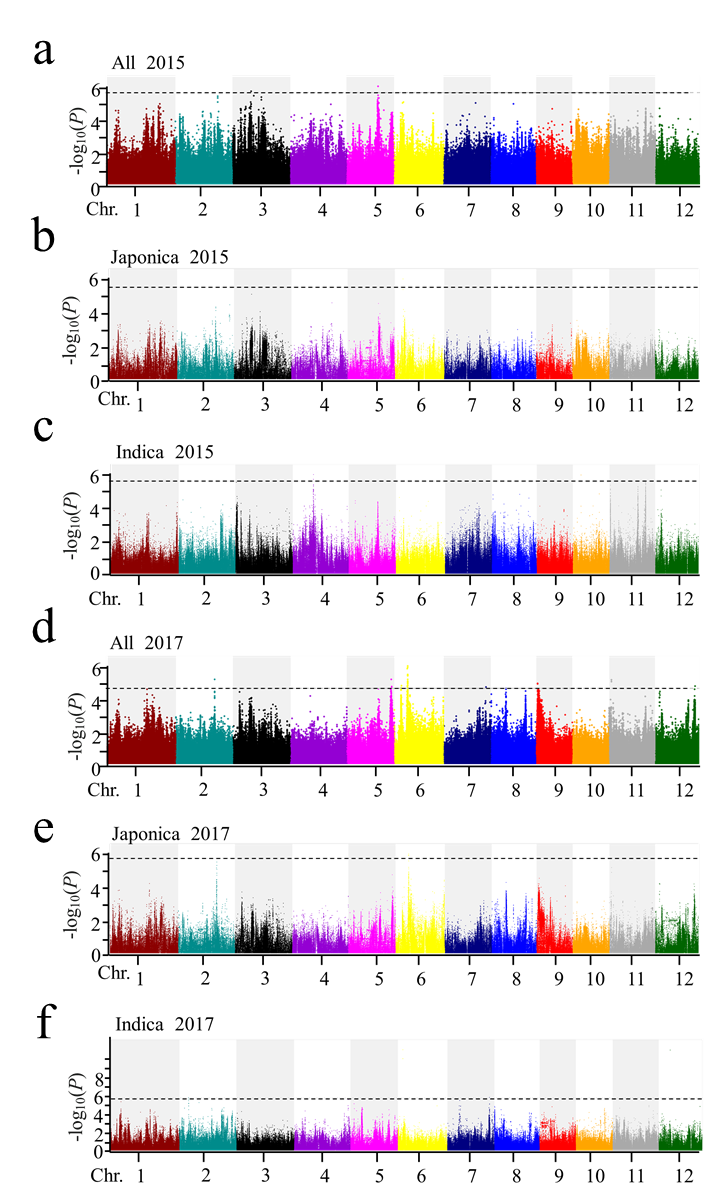


**Figure S5** Manhattan plots of GWAS for primary branch number in **a–c** 2015 and **d–f** 2017 for **a, d** all accessions; **b, e** japonica accessions; and **c, f** indica accessions. The black horizontal dashed line indicates the genome-wide significance threshold, ‑log_10_(*P*) > 5.69.


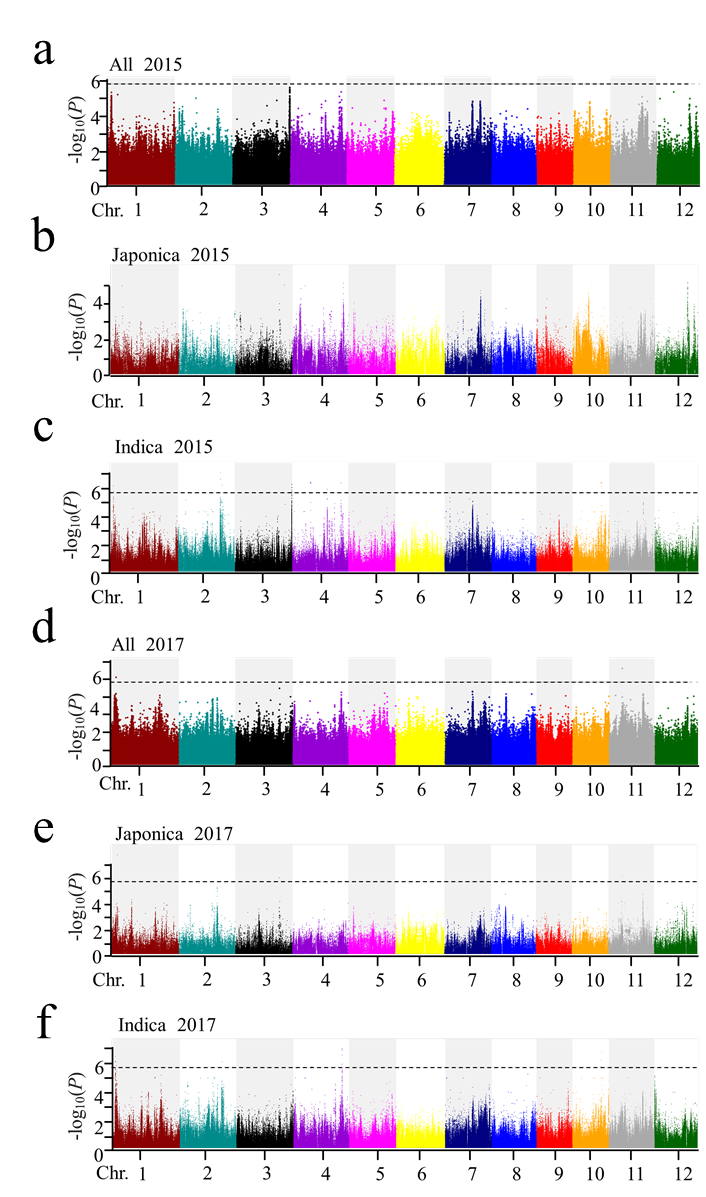


**Figure S6** Manhattan plots of GWAS for secondary branch number in **a–c** 2015 and **d–f** 2017 for **a, d** all accessions; **b, e** japonica accessions; and **c, f** indica accessions. The black horizontal dashed line indicates the genome-wide significance threshold, ‑log_10_(*P*) > 5.69.


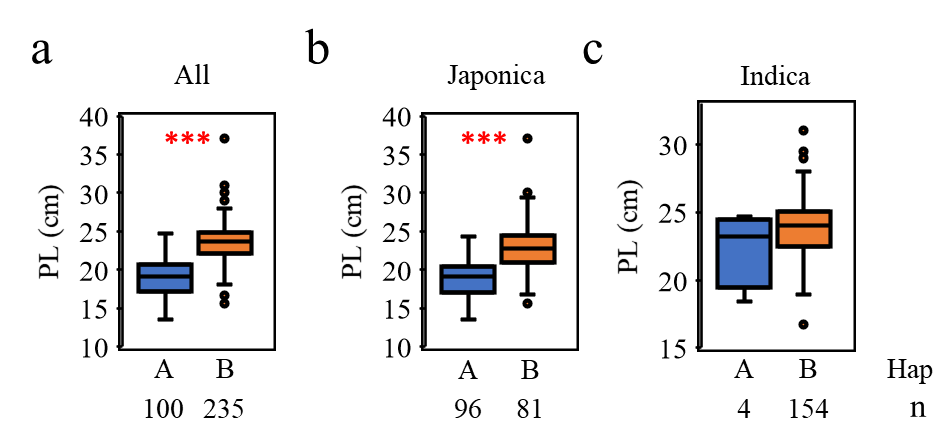


**Figure S7** Panicle length (PL) in the two haplotypes of *OsGRRP* in **a** all accessions, **b** japonica accessions, and **c** indica accessions in 2015. Number of accessions (n) of each haplotype (Hap) in each panel given under the x-axis. Boxes show median, and upper and lower quartiles. Whiskers extend to 1.5× the interquartile range, with any remaining points indicated with dots. *** *P*<0.001 (Welch two sample *t*-test).


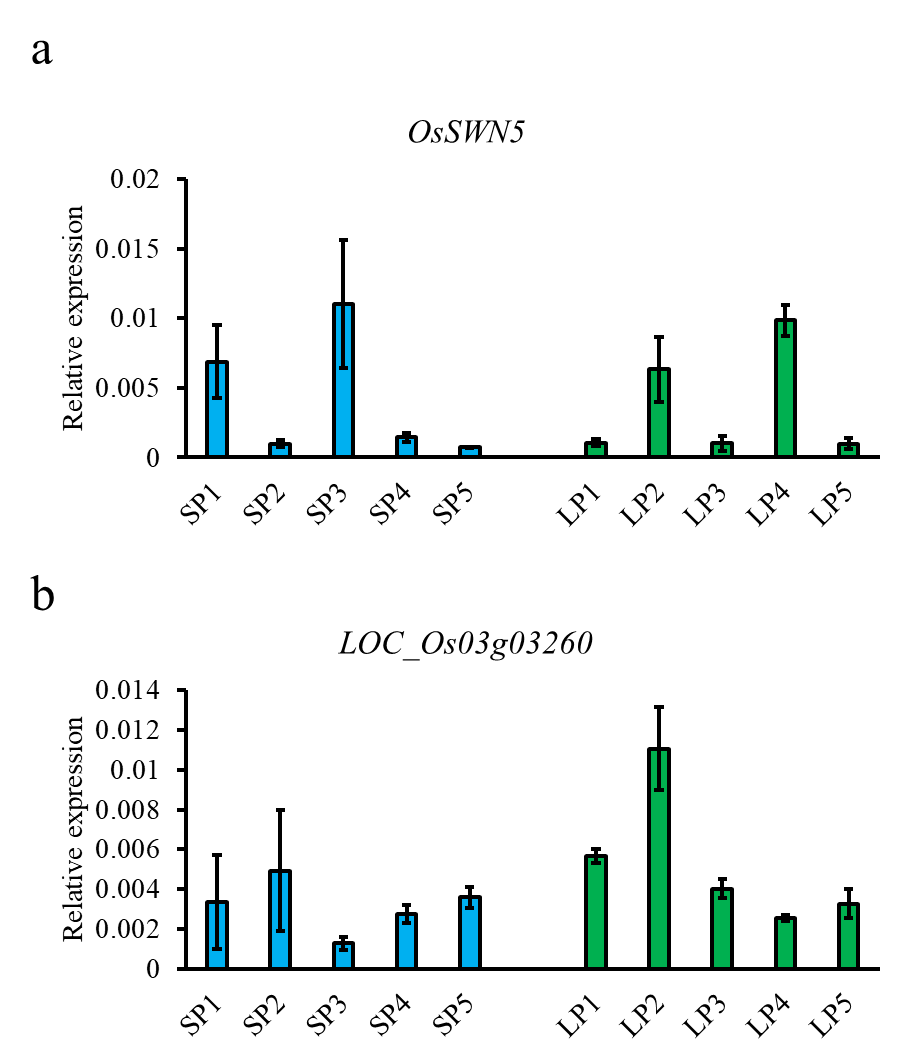


**Figure S8** Relative expression of **a** *OsSWN5* and **b** *LOC_Os03g03260* in short panicle (SP) and long panicle (LP) accessions. SP1: IRIS_313-8085; SP2: IRIS_313-8096; SP3: IRIS_313-8099; SP4: IRIS_313-8195; SP5: IRIS_313-8048; LP1: IRIS_313-8903; LP2: B199; LP3: IRIS_313-9505; LP4: IRIS_313-7993; LP5: IRIS_313-799
